# Gender specific mental health among adolescents in Northern Italy: a cross-sectional study

**DOI:** 10.3389/fpubh.2025.1705580

**Published:** 2026-01-16

**Authors:** Verena Barbieri, Giuliano Piccoliori, Adolf Engl, Christian J. Wiedermann

**Affiliations:** 1 Institute of General Practice and Public Health, Claudiana College of Health Professions, Bolzano, Italy; 2 Department of Public Health, Medical Decision Making and Health Technology Assessment, University of Health Sciences, Medical Informatics and Technology, Hall in Tirol, Tyrol, Austria

**Keywords:** adolescents, mental health, gender differences, post-COVID, school stress, problematic internet use, health literacy, anxiety

## Abstract

**Background:**

Recent studies show that the prevalence of many adolescents' mental health problems worsened during the pandemic, and improved recently, but is still higher than before the pandemic. The assessment of post-pandemic gender-specific mental health issues and their relation to global crises are of current interest.

**Methods:**

A repeated cross-sectional design screened self-reported mental health in terms of anxiety, depression, and general mental disorders of 11–19 years aged students in South Tyrol, North Italy four times between 2021 and 2025 using standardized instruments. The study was representative for age and gender, screening was done for the whole population, not regarding the same participants in all four surveys.

**Results:**

A total of 1,471 questionnaires were evaluated in 2025, nearly 11% of the participants were screened positively for depression and 28% for anxiety. While percentages were the same in all 4 years for anxiety, we found a decreasing trend for depression. About 40% of females in 2025 were screened positively for at least one mental health problem, for males the percentage was about 27%. The difference was significant. About 45% of the positively screened cases were screened positively by more than one instrument, while the percentage was 35% for males. The most important, non-gender-dependent associated factors were school stress, crises-related stress, and problematic Internet use. Low/medium health literacy and single parenthood were predictors of mental health problems among girls. Less sleep and less physical activity were significant factors.

**Discussion:**

Gender specific mental health problems in adolescents are still high post-pandemically. Mental health interventions, focusing on gender-specific requirements, are needed in the light of new global crises. Educational settings should integrate preventive strategies and health literacy programs based on existing experiences and continually collected new data.

## 1 Introduction

Mental health problems among adolescents, especially anxiety and depression, have increased substantially over the past two decades in many Western countries, with a marked increase during the COVID-19 pandemic. Prior to the pandemic, 13%−20% of children and adolescents worldwide were affected by mental health disorders (1). During the pandemic, self-reported anxiety and depression rose significantly across countries and have yet to return to pre-pandemic levels (2–4). In Germany, for instance, elevated Strengths and Difficulties Questionnaire (SDQ) scores rose from 18 to 31% in January 2021 and remained at 22% in 2024, while elevated anxiety scores increased from 14.9 to 30% and have since declined only slightly to 23% (5, 6).

Several studies have documented a rising trend in mental health issues among adolescents before the pandemic. In the United States, the prevalence of depression increased from 8.7% in 2005 to 15.8% in 2019 (7, 8). These gender differences were confirmed in UK and European studies, which showed that mental health outcomes worsened more markedly for girls than for boys (9, 10).

Italian data show similar patterns: prior to the pandemic, 17.8% of adolescents reported symptomatic SDQ scores, and 20.8% had depressive symptoms (11). Psychiatric admissions for adolescents increased by 200% over 2 years in the pandemic, with a stronger impact on females (12). A longitudinal survey in Italy, Spain, and Portugal found a sharp rise in mental health problems early in the pandemic, followed by partial improvements by 2023 (13).

Recently, mental health research has expanded its focus to include not only the effects of pandemics but also broader global crises, such as climate change, armed conflicts, and economic uncertainty. Findings from the COPSY-Germany surveys suggest that adolescents' mental health is sensitive to cumulative crisis exposure, with increased distress related to economic hardship, war-related concerns, and ongoing climate worries (14, 15).

South Tyrol, a bilingual region in Northern Italy with distinct German- and Italian-speaking school systems, offers a unique context for examining adolescent mental health. The region has a strong public health infrastructure and has participated in repeated youth health surveys since 2021 (16, 17), called COP-S (COrona and Psyche South Tyrol) studies. This allows not only for cross-sectional analysis but also for tracking mental health trends over several years in a consistent population sample. The sample mirrors western high-income countries' society and offers a controlled context for examining the roles of digital media use, and perceived social support. Problematic Internet use has been linked to a range of mental health problems, especially in male adolescents (18, 19), and excessive digital engagement during the pandemic intensified risks related to social comparison, fear of missing out, and exposure to negative content (20, 21). School-related stress, insufficient sleep, and lack of physical activity have been identified as key risk factors for depression (22, 23). Meanwhile, the roles of parental education, family structure, and migration background continue to be debated (24, 25).

This study aims to:

1. Assess the current state of adolescent mental health in South Tyrol in 2025.
2. Examine how the prevalences of mental health outcomes co-occurred between 2021 and 2025 analyzing four consecutive cross-sectional surveys.
3. Analyze gender-specific differences in mental health profiles.
4. To associate the most prevalent post-pandemic mental health issues to relevant demographic and lifestyle factors.

By offering a detailed, gender-sensitive analysis of adolescent mental health in a clearly defined region, this study aims to contribute to a deeper understanding of post-pandemic adolescents' mental health issues and to offer practical insights for prevention and policy at regional and international levels. The use of standardized, internationally validated measures, the analyses of lifestyle parameters and the large data set can support the applicability of the results to other populations from high income countries.

## 2 Materials and methods

### 2.1 Study design and sample

The survey was conducted for the fourth time after spring 2021, 2022 and 2023 (16, 17) using a repeated cross-sectional design. Participants were independently collected, and thus, not the same in the four surveys. The methodology aimed to provide a comparable approach in all four investigations to reflect the nature of the pandemic's impact on youth mental health. The goal was to provide up-to-date snapshots of the mental health among the region's youth and contribute to a broader understanding of the medium- and long-term trends of the pandemic. No weights were applied to the data, since data were representative for age and gender of the youth and there were no significant differences between the demographic parameters of the four survey years.

This study was designed as an anonymous online survey to assess the mental health of children and adolescents in South Tyrol. The questionnaire was accessible from 17 March 2025 until 13 April 2025 using the SoSci Survey Software, Version 3.2.46 (SoSci Survey GmbH, Munich, Germany).

The present study presents self-reported data from participants aged 11–19 years. They were recruited from all schools across the province by contacting their parents via email and sending a link to the online questionnaire. Informed consent was obtained from the parents and adolescents. Adolescents could complete self-report forms after their parents had completed the proxy version. Due to the anonymous online character of the questionnaire, we are not conscious of whether adolescents completed the questionnaire independently or with parental oversight. The questionnaire was distributed to over 40,000 families and was available in German and Italian language. All standardized instruments used in the survey were available in both languages and are referenced in the Section 2.

This study was conducted in accordance with the Declaration of Helsinki and approved by the Ethics Committee of the Autonomous Province of Bolzano, Italy (protocol code 52-2021 on April 21, 2021; protocol code 0304767 on 16 March 2022; protocol code 16-2023 on 15 March 2023; protocol code 11-2025 on 19 February 2025). Informed consent was obtained from all participating parents/guardians and adolescents.

### 2.2 Sociodemographic and lifestyle measures

Sociodemographic variables included age and gender of the children and parents, information about urban/rural residency, single parenthood, migration background, and parental educational attainment (26).

The actual number of days with more than 1 h of sports activity per week (from 1, “0 days” to 8, “7 days”) was recorded and dichotomized into 0 = 0–2 days a week and 1 = 3 or more days a week.

The multidimensional scale of perceived social support (MSPSS) assessed on a 12-item scale perceived social support from family, friends, and others (27). Answers were available on a seven-point Likert scale (1 = strongly disagree, …, 7 = strongly agree). The overall sum score was categorized according to the method described by Zimet (27).

Problematic Internet use was assessed using the German (28) and Italian (29) versions of the GPIUS-2 (generalized problematic internet use scale 2). The scale is composed of 15 questions with answers on an eight-point Likert scale (1 = “definitely disagree,” …,8 = “Definitely agree”). The total sum score was categorized according to the method described by Machimbarrena et al. (30).

The health literacy (HL) of adolescents was assessed using the Health Literacy for School-Aged Children (HLSAC) of the Health Behavior in School-aged Children (HBSC) study (31). Ten questions on a 4-point Likert scale ranging from 1 = “not at all true” to 4 = “absolutely true” led to a sum score ranging from 10 to 40, which was classified into low, moderate, and high HL.

Parents and adolescents were asked about their own perceived burden regarding the pandemic, climate change, wars in Ukraine and the Middle East, price increases, and natural catastrophes (items on a five-point Likert scale ranging from 1, “not at all” to 5, “completely”). The Questions and scaling were used according to the question regarding the burden due to the pandemic in (16, 17, 32, 33). An overall dichotomous indicator was calculated, with 1 = at least one of the five burdens is rated with 4 or five and 0 = none of the five burdens is rated with 4 or 5.

### 2.3 Mental health

The self-reported aspects of mental health in adolescents were assessed using the following screening instruments All screening instruments are validated and reliable measures to give indications of signs of mental health symptoms, but cannot substitute a clinical diagnosis.

Screen of child anxiety related emotional disorders (SCARED): the generalized anxiety disorder (GAD-9) subscale asked nine questions, such as “I worry about other people liking me.” and used a three-point response scale (0, “not or hardly true” to 2, “very or often true”) and is recommended to be applied in children and adolescents and validated for the corresponding cultural background (34, 35). A total score of nine or more was used as official cutoff. Good validity and reliability of GAD-9 subscale have been demonstrated and discussed in (34, 35), and originally in (36). This design was used in all four studies and is based on the design of (32, 33).

Patient health questionnaire-2 (PHQ-2): this questionnaire asked two questions regarding depression on a four-point Likert scale (from 0, “nearly never” to 3, “nearly every day”). It is recommended to be applied in adolescents aged 12 and older and validated for the corresponding cultural background (37, 38). A total score of three or more was used as official cutoff.

Strengths and difficulties questionnaire (SDQ): the SDQ assessed the mental wellbeing of adolescents across five dimensions, each consisting of five questions, each on a three-item response scale (0 = not true; 1 = somewhat true; 2 = certainly true): emotional problems, conduct problems, hyperactivity/inattention, peer relationship problems, and prosocial behavior. The questionnaire is designed for children and adolescents, and validated versions are available international comparisons (39). The total problem score was calculated from the former four subscales and could achieve values between 0 and 40. It was categorized according to official cut-offs into noticeable/abnormal, borderline, and normal categories. Higher scores indicate more mental health problems. In the Section 3, the categorization was dichotomized, summarizing the noticeable/abnormal and borderline cases as elevated.

### 2.4 Data analysis

Descriptive statistics of metric variables are presented as means (M) and standard deviations (SD), and categorical variables as absolute counts and percentages. Confidence intervals for the rates were computed, assuming a binomial distribution. Phi-coefficient indicated the effect size between dichotomous variables. Differences between groups were assessed using chi-square tests. Metric and ordinal variables were compared using the Mann-Whitney *U* test, and correlations were determined using Kendall tau b and point biserial coefficient. Correlations between all factors were measured to avoid redundancy.

Exact binomial confidence intervals for prevalences were calculated to make comparisons between years.

Reliability analysis of the sum scores was performed using Cronbach's alpha.

The main outcomes were dichotomized variables indicating above-threshold scores of depression (PHQ-2), anxiety (GAD-9), and generalized mental health disorders (SDQ), with 1 indicating ≥ threshold and 0 indicating < threshold.

To model the relationship between above-threshold cases and correlated variables, stepwise forward multiple logistic regression was performed, presenting significant odds ratios (OR) with 95% confidence intervals (CI) and corresponding *p*-values. Hosmer-Lemeshow statistics and Nagelkerke's *R*^2^ assessed goodness of fit. Of two variables having a correlation coefficient < 0.3 only one was included. We conducted multicollinearity assessment calculating the variance inflation factor.

Bujang et al. (40) recommended a minimum sample size of at least *n* = 500 for observational studies with large populations. It is also suggested to use formula *n* = 100 + i × 50, where “i” is the number of independent variables. Thus, with maximal nine independent variables in the model, the total required sample size is *n* = 100 +9 × 50 = 550.

The significance levels were set at 0.05 (^*^), 0.01 (^**^), and 0.001 (^***^), n.s. indicates “not significant.” All statistical analyses were performed using IBM SPSS Statistics for Windows (version 25.0; IBM Corp., Armonk, N.Y., USA).

## 3 Results

The overall response rate was about 23%. After data cleaning, ~80% of the data were analyzable. Out of the 4,498 analyzable parental questionnaires, 2,554 adolescents between 11 and 19 years confirmed their participation and 1,471 (57,6%) of these self- reports were complete regarding SDQ, GAD-9, and PHQ-2 answers. In addition to the results of the complete cases, in Supplementary material 1, we provide a comparison of parental answers for both groups, the group of complete and the group of sparsely completed self-reports.

The data were representative for gender and age of adolescents and the educational status of the parents according to the provincial institute of statistics ASTAT. Approximately 29% of the participating adolescents resided in urban areas, and ~9% had a migration background.

Cronbach's alpha was 0.899 for GAD-9, 0.728 for PHQ-2, 0.976 for MSPSS, 0.925 for GPIUS-2, 0.892 for HLSAC and 0.842 for HBSC.

Of the 1,471 cases, 732 males, 738 females and 1 diverse, 491 [33.3% (31.01; 35.83%)] were above threshold for at least one of the three mental health questionnaires, with girls showing a percentage of 39.8% [36.4; 43.4%] and boys 26.7% [23.7; 31.1%]. The diverse participant was above threshold for all three questionnaires but was no longer included in analyses. A total of 410 [27.9% (25.6; 30.2%)] patients were above threshold for GAD-9, of whom 268 were female. A total of 204 [13.9% (12.2; 15,7%)] adolescents were above threshold for SDQ, of whom 120 were female. An elevated PHQ-2 score was found in 158 [10.7% (9.3; 12.4%)] participants, including 98 females.

### 3.1 Prevalences of GAD-9 and PHQ-2 between 2021 and 2025

The COP-S studies in 2021, 2022, and 2023 even asked the GAD-9 and PHQ-2 questionnaires in self-reports. Due to the cross-sectional and anonymous nature of the surveys, the participants were not the same, and the results of this section cannot be considered longitudinal data but are representative of the age and gender of adolescents in the corresponding years.

In 2021, 1,867 adolescents completed the survey, with 15.4% [13.9; 17.1%] and 27.1% [25.2; 29,2%] of them having elevated depression and anxiety scores, respectively. In 2022, 2,070 adolescents completed the survey, with corresponding percentages of 13.7% [12.4; 15.4%] and 27.0% [25.2; 29.1%], respectively. In 2023, after the pandemic, 1,557 complete answers showed a rate of 11.9% [10.3; 13,5%] of elevated depressive scores, while 27.7% [25.5; 30.0%] showed an elevated score of anxiety.

**Figure 1:**
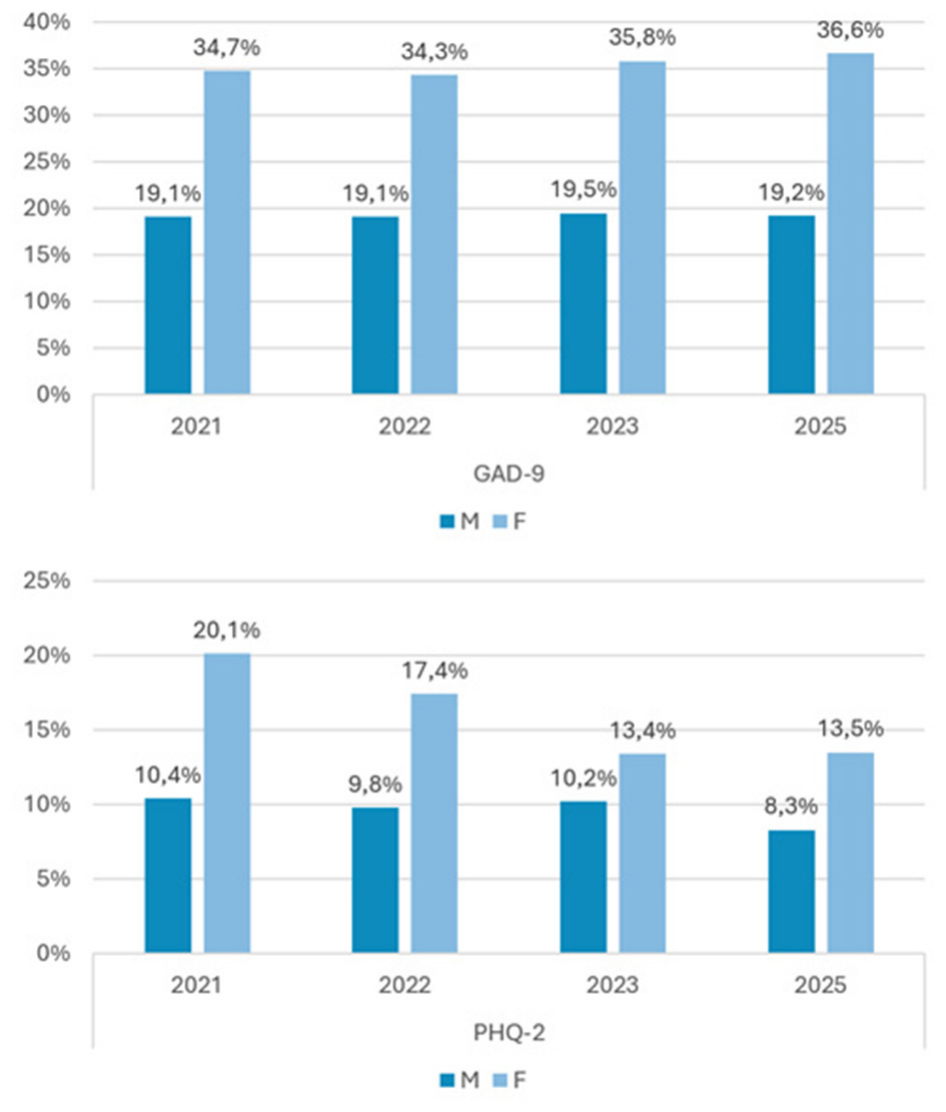
Prevalences of anxiety and depression elevated scores in 2021, 2022, 2023, and 2025 for males and females aged 11–19.

The prevalences per gender are presented in Figure 1. Females suffered from anxiety significantly more often than males during the pandemic and today (2021: phi = 0.176; *p* < 0.001; 2022: phi = 0.176; *p* < 0.001; 2023: phi = 0.185; *p* < 0.001; 0.189; *p* < 0.001). For depression, females suffered significantly more often than males in 2021 (phi = 0.137; *p* < 0.001), 2022 (0.114; *p* < 0.001), 2023 (0.050; *p* = 0.043), and 2025 (0.082; *p* = 0.002).

The exact confidence intervals for the prevalence of elevated GAD-9 did not reveal any significant differences between the years. For elevated PHQ-2, we found a significant decrease in prevalence of female participants in 2023 [13.4 (11.1; 16.0)] and 2025 [13.5 (10.9; 15.9)] compared to 2021 [20.1 (17.8; 22.9)] and 2022 [17.4 (15.2; 19.9)]. Even for males, the percentage for 2021 (10.4) lay outside the confidence interval for 2025 [8.3 (6.2; 10.3)].

### 3.2 Combinations of positively screened questionnaires in 2025

**Figure 2:**
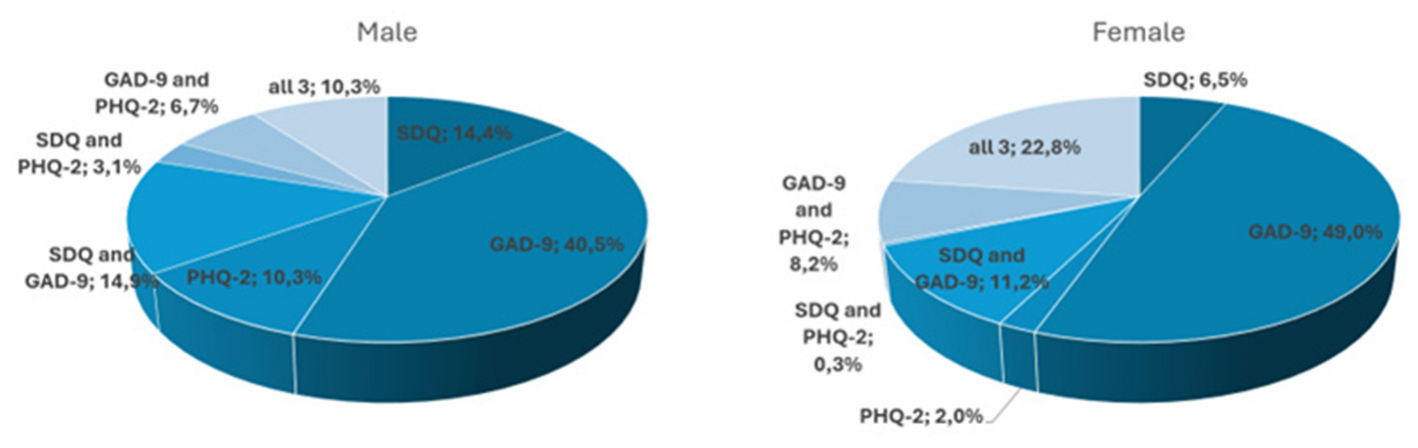
Single and multiple elevated scores per gender.

Of the 490 positively screened cases, 60.4% were screened positive for only one mental health issue (45.5% GAD-9, 9,6% SDQ and 5.3% PHQ-2). 12.7% were screened positive for both, SDQ and GAD-9, 1.4% were screened positive for SDQ and PHQ, and 7.6% were screened positive for GAD-9 and PHQ-2. In 18.0%, all three questionnaires returned positive screening results. Figure 2 shows the distribution of combinations of positive screened questionnaires by gender. We found that males were more often above cutoff for SDQ and/or PHQ-2, but not for GAD-9 (27.8%), while we met this pattern only in 8.8% of female participants.

### 3.3 Demographics and lifestyle parameters

Demographic and lifestyle parameters are presented in Table 1 for the whole sample. *p*-values indicate whether positively screened cases differ significantly from not positively screened cases regarding these parameters. The groups of adolescents with elevated SDQ, GAD-9, or PHQ-2 scores, had significantly more often female gender, a single parent, an elevated GPIUS-2, a lower HLSAC, lower MSPSS, did less sports, felt more school burdened, and more burdened about global crises. Adolescents with elevated GAD-9 scores were more likely to be urban residents. All elevated scores were significantly associated with older age and less sleep.

**Table 1:** Demographic and lifestyle parameters for SDQ, GAD-9 and PHQ-2 scores above threshold.

**Demographics**	**Total (*N* = 1,470)**	**SDQ (*N* = 204) above threshold**	**SCARED (*N* = 410) above threshold**	**PHQ-2 (*N* = 158) above threshold**
		***p*** **-value** ^**#**^	***p*** **-value** ^**#**^	***p*** **-value** ^**#**^
Age		< 0.001	< 0.001	< 0.001
Hours of sleep		< 0.001	< 0.001	< 0.001
	**%**	***p*** **-value** ^*^	***p*** **-value** ^*^	***p*** **-value** ^*^
Female	50.17	0.008	< 0.001	< 0.001
Single parent	11.95	< 0.001	< 0.001	0.016
German questionnaire language		0.081	0.174	0.294
Migration background	8.53	0.073	0.692	0.094
Urban residence	28.69	0.361	0.048	0.153.
Low educational level	19.23	0.0.495	0.0.938	0.696
Elevated GPIUS-2	26.85	< 0.001	< 0.001	< 0.001
Sport three times a week	68.83	< 0.001	< 0.001	< 0.001
High school burden	40.33	< 0.001	< 0.001	< 0.001
**HLSAC**				
Low	11.09	< 0.001	0.002	0.017
Middle	66.64			
High	22.26			
Burden of global crises	40.67	< 0.001	< 0.001	< 0.001
**MSPSS**				
Low	6.38	< 0.001	< 0.001	< 0.001
Moderate	10.48			
High	57.14			

^*^Chi-square test comparing cases ≥ threshold to cases < threshold.

^#^Man-Whithney test comparing elevated cases (≥ threshold) to not elevated cases (< threshold).

Parental educational level and migration background were not associated with elevated scores. SDQ, GAD-9 and PHQ-2 above threshold prevalences did not differ between questionnaire language.

### 3.4 Logistic regression for gender-related elevated GAD-9 score

For males, elevated GAD-9 score was significantly correlated with high MSPSS (−0.143^***^), elevated GPIUS-2 (0.296^***^), number of hours of sleep (−0.117^***^), burden of global crises (0.204^***^), school burden (0.240^**^), at least 3 h of sports a day (−0.156^***^), and age (0.104^**^), but not with high HLSAC, single parenthood, urban residency, and migration background.

For females, elevated GAD-9 score was significantly correlated with high MSPSS (−0.146^***^), high HLSAC (−0.158^***^), schools' burden (0.358^***^), burden of global crises (0.198^***^), single parenthood (0.126^***^), elevated GPIUS-2 (0.316^***^), number of sleeping hours (−0.211^***^), at least 3 days of sports a week (−0.174^***^), age (0.143^***^), and urban residency (0.082^*^), but not with migration background.

All independent variables were only weakly correlated between each other (Kendall tau-b and point biserial coefficients < 0.3).

Logistic regression models for both genders demonstrated a non-significant (*p* > 0.1) Hosmer-Lemeshow statistic, thus having a reliable goodness of fit. Nagelkerke's *R*^2^, odds ratios with 95% confidence intervals (CI), and corresponding *p*-values are presented in Table 2. The variance inflation factor was < 1.2 in both models, thus no multicollinearity problem was found.

**Table 2:** Odds ratios with 95% confidence intervals of logistic regression models for elevated GAD-9 scores for males and females.

**Parameters**	**Male**		**Female**	
Nagelkerke's *R*^2^	0.24		0.32	
Hosmer Lemeshow	0.833		0.763	
	**OR [95% CI]**	***p*** **-value**	**OR [95% CI]**	***p*** **-value**
Hours of sleep	0.69 [0.56; 0.84]	< 0.001	0.69 [0.56; 0.84]	< 0.001
Single parent			1.77 [1.01; 3.09]	0.045
Urban residence				n.s.
Elevated GPIUS2	2.75 [1.87; 4.11]	< 0.001	2.46 [1.64; 3.68]	< 0.001
Sport three times a week	0.63 [0.44; 0.91]	0.015	0.60 [0.41; 0.87]	0.007
High school burden	3.72 [2.59; 5.36]	< 0.001	3.48 [2.33; 5.06]	< 0.001
HLSAC high			0.53 [0.32; 0.87]	0.012
Burden of global crises	1.66 [1.15; 2.39]	0.006	1.70 [1.01; 3.09]	0.045
MSPSS high	0.58 [0.36; 0.94]	0.026		n.s.
Constant	7.44	0.019		n.s.

n.s., not significant.

The variable was not included in the stepwise forward regression model.

For males and females, fewer hours of sleep, an elevated GPIUS-2, and high school burden were highly significant associated with elevated GAD-9 scores. Even less sports and the burden of global crises were significantly associated with elevated GAD-9 score. For males, low/medium MSPSS was a significant independent variable. For females, single parenthood and low/medium HLSAC were highly associated with elevated GAD-9 score.

Nagelkerke'S *R*^2^ was about 0.32 for females, indicating a moderate explanation of the variance, while it was about 0.24 for males, indicating a lower explanation of the variance, thus showing a weaker relationship between outcome and associated variables for males than for females.

### 3.5 Logistic regression for elevated SDQ and PHQ-2 for males

Analyses of section 3.2 have shown that 27.8% of all male cases with above-threshold scores were not above-threshold for GAD-9. Thus, we analyzed these cases separately in logistic regression models.

For males, elevated PHQ-2 was significantly correlated with schools' burden (0.172^***^), burden of global crises (0.102^**^), elevated GPIUS-2 (0.159^***^), number of hours of sleep (−0.139^***^), and age (0.138^***^), but not with high MSPSS, high HLSAC, single parenthood, at least 3 days of sports a week, urban residency, and migration background. Elevated SDQ was significantly correlated with high MSPSS (−0.236^***^), high HLSAC (−0.096^*^), school burden (0.245^***^), elevated GPIUS-2 (0.242^***^), hours of sleep (−0.069^*^), at least three times sports a week (−0.102^***^), age (0.063^*^), and burden of global crises (0.141^***^), but not with single parenthood, urban residency, and migration background. Age and hours of sleep were correlated (−0.506^***^). Therefore, age was not included in the models. All independent variables were weakly correlated between each other (Kendall tau-b and point biserial coefficients < 0.3).

Both models demonstrated a non-significant (*p* > 0.1) Hosmer-Lemeshow statistic, thus having a reliable goodness of fit. Nagelkerke's *R*^2^, odds ratios with 95% confidence intervals (CI), and corresponding *p*-values are presented in Table 3. The variance inflation factor was < 1.2 in both models, thus no multicollinearity problem was found.

**Table 3:** Odds ratios with 95% confidence intervals of the logistic regression model for elevated SDQ and PHQ-2 score for males.

**Parameters**	**SDQ**		**PHQ-2**	
Nagelkerke's *R*^2^	0.28		0.13	
Hosmer Lemeshow	0.426		0.134	
	**OR [95% CI]**	***p*** **-value**	**OR [95% CI]**	***p*** **-value**
Hours of sleep		n.s.	0.60 [0.44; 0.81]	0.001
Elevated GPIUS2	3.75 [2.15; 6.53]	< 0.001	2.38 [1.30; 4.36]	0.005
Sport three times a week		n.s.		
High school burden	5.02 [2.76; 9.13]	< 0.001	2.20 [1.19; 4.06]	0.006
HLSAC high	0.407 [0.17; 1.0]	0.05		
Burden of global crises		n.s.		n.s.
MSPSS high	0.224 [0.12; 0.40]	< 0.001		
Constant	0.10	< 0.001		n.s.

n.s., not significant.

The variable was not included in the stepwise forward regression model.

For elevated SDQ scores, as well as for elevated PHQ-2 scores, elevated GPIUS-2 and high school burden were found to be significant associates. Elevated SDQ scores were significantly associated with low/medium HLSAC and low/medium MSPSS scores, while elevated PHQ-2 scores were significantly associated with less hours of sleep. Nagelkerke'S *R*^2^ was about 0.28 for SDQ, indicating a moderate explanation of the variance, while it was about 0.13 for PHQ, indicating a low explanation of the variance, thus showing a weak relationship between outcome and associated variables for PHQ-2 and moderate relationship for SDQ in males.

## 4 Discussion

The post-pandemic investigation om mental health in South Tyrolean adolescents has led to some interesting insights in the light of gender differences and associated factors. As the pandemic officially in Italy ended on 5th of May 2023, this study shows that 2 years after the pandemic, mental health problems remain a major issue among adolescents in South Tyrol. One in three adolescents showed above threshold scores in at least one of the three mental health screening tools, and girls were significantly more affected than boys were. Notably, girls were more likely to report combinations of above threshold scores. The results suggest significant associations between mental health problems and perceived school pressure, global crises, low social support, and problematic Internet use.

Our results show a decreasing trend from 2021 to 2025 in the prevalence of signs of depression, but no change in the prevalence of signs of anxiety. These results align with international results from a systematic review of 2022 (41) and actual results of similar studies in Europe (6, 11, 42), showing that symptoms of depression have returned to pre-pandemic percentages, but elevated SDQ and especially symptoms of anxiety remained higher than before the pandemic, especially for girls (10). Our findings extend these observations by providing more recent data from 2025 and confirming the persistence of gender mental health disparities, especially regarding overlapping symptoms. The predominance of anxiety signs in girls, nearly in all cases that show elevated SDQ or PHQ-2 scores, is an important result that must be interpreted with caution. Factors associated with symptoms of anxiety, like hours of sleep, high school burden or problematic internet use may overlap with factors that are associated to SDQ and PHQ-2 and may obscure them when not analyzed with caution. Targeted gender specific monitoring and screening are needed.

Global crises are related to elevated anxiety scores in both males and females, while there were no relations with males' above-cutoff depression scores and SDQ scores. These results align with (6). It is a challenging question to investigate adolescents' post-pandemic mental health considering global crises, and further investigations are needed to understand the direction of this association.

Single parenthood was more associated to problems in females, whereas low/medium social support was a predictor of problems in males. The problem of single parenthood during the pandemic is discussed in (24) and requires further intensive and detailed analyses to identify possible solutions. Migration background and parental education were not associated with self-reported mental health issues. Missing evidence was confirmed in (25), but the results should be interpreted with caution in the context of possible unmeasured mental health parameters or reporting bias. Finally, good knowledge of one's own health was correlated with mental health issues especially for girls. Further, more detailed investigations, especially in mental health literacy, are needed to address interventions.

Despite the gender gap, problematic Internet use, too little sleep, little physical activity, and school burden predicted mental health problems. Responsible use of digital media and targeted approaches to address school issues can help reduce mental health problems. Similar results regarding digital media use were found in other studies (21), where the authors suggested replacing screen time with exercise and making adolescents more aware of adverse mechanisms such as social comparison, fear of missing out, and exposure to negative content. Pre-pandemic HBSC studies in Italy found elevated psychological health complaints positively associated with higher school pressure (43). The results are in line with the 24-h movement guidelines (44) recommending a minimum of 60 min/day of moderate-to-vigorous physical activity, no more than 2 h/day of recreational screen time, and 8–11 h/night of sleep depending on age. These recommendations are simple and can easily be integrated into the daily lives of adolescents. An appropriate strategy could be implemented throughout educational settings. They can integrate health literacy programs, digital health education, screen time awareness, and stress regulation into everyday practice and promote psychosocial wellbeing as part of a broader preventive strategy. Further, even during school lessons guidelines can be applicated, by finding strategies to reduce screen use at school and introducing breaks and body movement after long periods of sitting in the same place. The implementation throughout schools has the advantage that all children and adolescents are reached. Programs should start already in elementary schools as preventive strategy, even involving parents. Implementation must be conducted by policy makers in an official and comprehensive way.

In this context, it is worth to mention, that there have not been found significant differences between the questionnaire languages German and Italian for SDQ, GAD-9 and PHQ-2 above threshold prevalences. This fact is interesting in the bilingual context of the region. While Italian speaking persons mainly live in urban areas, German speaking people prefer rural areas. Even the difference between urban and rural areas in adolescents' mental health problems was small or not significant. Thus, when discussing the mental health of adolescents in bilingual regions, we can affirm that the sanitary system is the same for both languages in our region as well as policy making. At this point, we only can state the importance of taking decisions about mental health care planning for all young people independently of their cultural background and to provide area-wide implementations of preventive strategies throughout German and Italian educational settings.

We can conclude, that mental health problems have partly decreased in the years after the pandemic, with different developments for males and females. The prevalence of elevated anxiety scores remained as high as during the pandemic. While the implementation of the 24-h movement guidelines in adolescents' daily lives can be a gender-independent intervention, targeted interventions for girls could be gender-specific health literacy programs and further monitoring of gender differences in mental health issues. Social support programs need to be implemented on a large scale with gender-sensitive interventions for adolescents with low support. Finally, the associations between adolescents' mental health and actual global crises are a new challenge that must be investigated and monitored in future surveys to address targeted prevention and intervention. The continuity of data collection in South Tyrol provides an exceptional opportunity to establish a regionally anchored early warning system for adolescent mental health that can be adapted to other health systems.

## 5 Strength and limitations

This study has several strengths. It is based on a large population-based sample, representative of age, gender, and parental education, and includes repeated cross-sectional data, allowing for the observation of trends beyond the acute pandemic period. The results provide a unique regional snapshot of adolescent mental health in South Tyrol, a province between the German and Italian cultural structures. The comparability of outcomes with other studies of high-income countries underscores that adolescents experience similar mental health challenges. However, this study has some limitations. All data were based on self-reports, which may be subject to reporting or self-selection bias or underestimation of mental health issues. The screening study did not include clinical assessments or diagnostic interviews, limiting the ability to draw conclusions regarding psychiatric disorders. For future research, a controlled survey comparing clinical cases to population-based screening could give more detailed information about symptom interpretation and implementation of systems for early detection. Furthermore, the absence of pre-pandemic baseline data restricts the interpretation of long-term mental health changes. Finally, in the study, the SDQ score is dichotomized, aggregating borderline and abnormal cases in one “elevated” group. This aggregation may lead to an overestimation of symptomatic SDQ cases.

## Data Availability

The datasets presented in this article are not readily available because data refer to participants younger than 18 years. Requests to access the datasets should be directed to Verena Barbieri verena.barbieri@am-mg.claudiana.bz.it.

## References

[1] Otto C Reiss F Voss C Wüstner A Meyrose AK Hölling H . Mental health and well-being from childhood to adulthood: design, methods and results of the 11-year follow-up of the BELLA study. Eur Child Adolesc Psychiatry. (2021) 30:1559–77. doi: 10.1007/s00787-020-01630-4 32918625 PMC8505294

[2] Saulle R De Sario M Bena A Capra P Culasso M Davoli M . School closures and mental health, wellbeing and health behaviours among children and adolescents during the second COVID-19 wave: a systematic review of the literature. Epidemiol Prev. (2022) 46:333–52. doi: 10.19191/EP22.5-6.A542.089 36384255

[3] Ludwig-Walz H Dannheim I Pfadenhauer LM Fegert JM Bujard M. Increase of depression among children and adolescents after the onset of the COVID-19 pandemic in Europe: a systematic review and meta-analysis. Child Adolesc Psychiatry Ment Health. (2022) 16:109. doi: 10.1186/s13034-022-00546-y 36587221 PMC9805372

[4] Wang S Chen L Ran H Che Y Fang D Sun H . Depression and anxiety among children and adolescents pre and post COVID-19: a comparative meta-analysis. Front Psychiatry. (2022) 13:917552. doi: 10.3389/fpsyt.2022.917552 35990058 PMC9381924

[5] Ravens-Sieberer U Devine J Napp AK Kaman A Saftig L Gilbert M . Three years into the pandemic: results of the longitudinal German COPSY study on youth mental health and health-related quality of life. Front Public Health. (2023) 11:1129073. doi: 10.3389/fpubh.2023.1129073 37397777 PMC10307958

[6] Kaman A Erhart M Devine J Napp AK Reiß F Behn S . Mental health of children and adolescents in times of global crises: findings from the longitudinal COPSY study from 2020 to 2024. Bundesgesundheitsblatt Gesundheitsforschung Gesundheitsschutz. (2025) 68:670–80. doi: 10.1007/s00103-025-04045-1 40293489 PMC12129870

[7] Vidal C Lhaksampa T Miller L Platt R. Social media use and depression in adolescents: a scoping review. Int Rev Psychiatry Abingdon Engl. (2020) 32:235–53. doi: 10.1080/09540261.2020.1720623 32065542 PMC7392374

[8] Wilson S Dumornay NM. Rising rates of adolescent depression in the United States: challenges and opportunities in the 2020s. J Adolesc Health Off Publ Soc Adolesc Med. (2022) 70:354–5. doi: 10.1016/j.jadohealth.2021.12.003 35183317 PMC8868033

[9] Mendolia S Suziedelyte A Zhu A. Have girls been left behind during the COVID-19 pandemic? Gender differences in pandemic effects on children's mental wellbeing. Econ Lett. (2022) 214:110458. doi: 10.1016/j.econlet.2022.110458 35345669 PMC8944107

[10] Madigan S Racine N Vaillancourt T Korczak DJ Hewitt JMA Pador P . Changes in depression and anxiety among children and adolescents from before to during the COVID-19 pandemic: a systematic review and meta-analysis. JAMA Pediatr. (2023) 177:567–81. doi: 10.1001/jamapediatrics.2023.0846 37126337 PMC10152379

[11] Donato F Triassi M Loperto I Maccaro A Mentasti S Crivillaro F . Symptoms of mental health problems among Italian adolescents in 2017-2018 school year: a multicenter cross-sectional study. Environ Health Prev Med. (2021) 26:67. doi: 10.1186/s12199-021-00988-4 34154531 PMC8216089

[12] Tavano S Caini S Sforzi I Silvestri C Voller F Pisano T. Mental health of children and adolescents before and during the COVID-19 pandemic: how did the lockdown modify psychiatric emergencies in Tuscany, Italy? J Clin Med. (2023) 12:4154. doi: 10.3390/jcm12124154 37373847 PMC10299620

[13] Amorós-Reche V Morales A Francisco R Delvecchio E Mazzeschi C Godinho C . Three years after the pandemic: how has the mental health of children and adolescents evolved? A Longitudinal Study in Italy, Spain, and Portugal. Span J Psychol. (2025) 28:e10. doi: 10.1017/SJP.2025.8 40211095

[14] Kaman A Erhart M Devine J Reiß F Napp AK Simon AM . Two years of pandemic: the mental health and quality of life of children and adolescents. Dtsch Arzteblatt Int. (2023) 120:269–70. doi: 10.3238/arztebl.m2023.0001 37482701 PMC10366960

[15] Cook R Nam X Weitzman M. Eco-anxiety and climate anxiety: bellwethers of the climate crisis's mental health impact on children and adolescents. J Dev Behav Pediatr JDBP. (2025) 46:e223–6. doi: 10.1097/DBP.0000000000001357 40232809

[16] Barbieri V Wiedermann CJ Piccoliori G Mahlknecht A Plagg B Ausserhofer D . Evolution of youth's mental health and quality of life during the COVID-19 pandemic in South Tyrol, Italy: comparison of two representative surveys. Child Basel Switz. (2023) 10:895. doi: 10.3390/children10050895 37238443 PMC10217242

[17] Barbieri V Piccoliori G Engl A Wiedermann CJ. Parental mental health, gender, and lifestyle effects on post-pandemic child and adolescent psychosocial problems: a cross-sectional survey in Northern Italy. Int J Environ Res Public Health. (2024) 21:933. doi: 10.3390/ijerph21070933 39063509 PMC11277222

[18] Xiang M Liu Y Yamamoto S Mizoue T Kuwahara K. Association of changes of lifestyle behaviors before and during the COVID-19 pandemic with mental health: a longitudinal study in children and adolescents. Int J Behav Nutr Phys Act. (2022) 19:92. doi: 10.1186/s12966-022-01327-8 35883177 PMC9321278

[19] El Asam A Samara M Terry P. Problematic internet use and mental health among British children and adolescents. Addict Behav. (2019) 90:428–36. doi: 10.1016/j.addbeh.2018.09.007 30579146

[20] Trott M Driscoll R Irlado E Pardhan S. Changes and correlates of screen time in adults and children during the COVID-19 pandemic: a systematic review and meta-analysis. EClinicalMedicine. (2022) 48:101452. doi: 10.1016/j.eclinm.2022.101452 35615691 PMC9122783

[21] Marciano L Ostroumova M Schulz PJ Camerini AL. Digital media use and adolescents' mental health during the covid-19 pandemic: a systematic review and meta-analysis. Front Public Health. (2021) 9:793868. doi: 10.3389/fpubh.2021.793868 35186872 PMC8848548

[22] Ruiz-Ranz E Asín-Izquierdo I. Physical activity, exercise, and mental health of healthy adolescents: a review of the last 5 years. Sports Med Health Sci. (2025) 7:161–72. doi: 10.1016/j.smhs.2024.10.003 39991129 PMC11846438

[23] Hill ED Kashyap P Raffanello E Wang Y Moffitt TE Caspi A . Prediction of mental health risk in adolescents. Nat Med. (2025) 31:1840–6. doi: 10.1038/s41591-025-03560-7 40044931 PMC12176513

[24] Naito T Tomata Y Otsuka T Tsuno K Tabuchi T. Did children in single-parent households have a higher probability of emotional instability during the COVID-19 pandemic? A nationwide cross-sectional study in Japan. Int J Environ Res Public Health. (2022) 19:4239. doi: 10.3390/ijerph19074239 35409920 PMC8998338

[25] Lievrouw S Myin-Germeys I Achterhof R. The mental health of European adolescents with vs. without a migration background (2013-2024)-a systematic review. Eur Child Adolesc Psychiatry. (2025) 34:1529–43. doi: 10.1007/s00787-024-02589-2 39467918

[26] Brauns H Scherer S Steinmann S. The CASMIN educational classification in international comparative research. In:Hoffmeyer-Zlotnik JHP Wolf C, editors. Advances in Cross-National Comparison: A European Working Book for Demographic and Socio-Economic Variables. Boston, MA: Springer US (2003). p. 221–44.

[27] Zimet G Dahlem N Zimet S Farley G. (1988). The multidimensional scale of perceived social support. J Personal Assess. 52:30–41. doi: 10.1207/s15327752jpa5201_2

[28] Barke A Nyenhuis N Kröner-Herwig B. The German version of the generalized pathological internet use scale 2: a validation study. Cyberpsychol Behav Soc Netw. (2014) 17:474–82. doi: 10.1089/cyber.2013.0706 24742070

[29] Casale S Primi C Fioravanti G. Generalized Problematic Internet Use Scale 2: Update on the Psychometric Properties Among Italian Young Adults. (2014). doi: 10.1037/t72193-000

[30] Machimbarrena JM González-Cabrera J Ortega-Barón J Beranuy-Fargues M Álvarez-Bardón A Tejero B. Profiles of problematic internet use and its impact on adolescents' health-related quality of life. Int J Environ Res Public Health. (2019) 16:3877. doi: 10.3390/ijerph16203877 31614899 PMC6843246

[31] Paakkari L Torppa M Mazur J Boberova Z Sudeck G Kalman M . A comparative study on adolescents' health literacy in Europe: findings from the HBSC study. Int J Environ Res Public Health. (2020) 17:3543. doi: 10.3390/ijerph17103543 32438595 PMC7277198

[32] Ravens-Sieberer U Kaman A Erhart M Devine J Schlack R Otto C. Impact of the COVID-19 pandemic on quality of life and mental health in children and adolescents in Germany. Eur Child Adolesc Psychiatry. (2022) 31:879–89. doi: 10.1007/s00787-021-01726-5 33492480 PMC7829493

[33] Ravens-Sieberer U Kaman A Erhart M Otto C Devine J Löffler C . Quality of life and mental health in children and adolescents during the first year of the COVID-19 pandemic: results of a two-wave nationwide population-based study. Eur Child Adolesc Psychiatry. (2023) 32:575–88. doi: 10.1007/s00787-021-01889-1 34636964 PMC8506100

[34] Weitkamp K Romer G Rosenthal S Wiegand-Grefe S Daniels J. German screen for child anxiety related emotional disorders (SCARED): reliability, validity, and cross-informant agreement in a clinical sample. Child Adolesc Psychiatry Ment Health. (2010) 4:19. doi: 10.1186/1753-2000-4-19 20591137 PMC2912250

[35] Crocetti E Hale WW Fermani A Raaijmakers Q Meeus W. Psychometric properties of the screen for child anxiety related emotional disorders (SCARED) in the general Italian adolescent population: a validation and a comparison between Italy and The Netherlands. J Anxiety Disord. (2009) 23:824–9. doi: 10.1016/j.janxdis.2009.04.003 19427168

[36] Birmaher B Brent DA Chiappetta L Bridge J Monga S Baugher M. Psychometric properties of the screen for child anxiety related emotional disorders (SCARED): a replication study. J Am Acad Child Adolesc Psychiatry. (1999) 38:1230–6. doi: 10.1097/00004583-199910000-00011 10517055

[37] D'Argenio P Minardi V Mirante N Mancini C Cofini V Carbonelli A . Confronto tra due test per la sorveglianza dei sintomi depressivi nella popolazione. Notiziario dell'Istituto Superiore di Sanità (2013) 26:i–iii.

[38] Schuler M Strohmayer M Mühlig S Schwaighofer B Wittmann M Faller H . Assessment of depression before and after inpatient rehabilitation in COPD patients: psychometric properties of the German version of the patient health questionnaire (PHQ-9/PHQ-2). J Affect Disord. (2018) 232:268–75. doi: 10.1016/j.jad.2018.02.037 29499510

[39] Goodman R. Psychometric properties of the strengths and difficulties questionnaire. J Am Acad Child Adolesc Psychiatry. (2001) 40:1337–45. doi: 10.1097/00004583-200111000-00015 11699809

[40] Bujang MA Sa'at N Sidik TMITAB Joo LC. Sample size guidelines for logistic regression from observational studies with large population: emphasis on the accuracy between statistics and parameters based on real life clinical data. Malays J Med Sci. (2018) 25:122–30. doi: 10.21315/mjms2018.25.4.12 30914854 PMC6422534

[41] Theberath M Bauer D Chen W Salinas M Mohabbat AB Yang J . Effects of COVID-19 pandemic on mental health of children and adolescents: a systematic review of survey studies. SAGE Open Med. (2022) 10:20503121221086712. doi: 10.1177/20503121221086712 35371484 PMC8972920

[42] Pedrini L Meloni S Lanfredi M Ferrari C Geviti A Cattaneo A . Adolescents' mental health and maladaptive behaviors before the Covid-19 pandemic and 1-year after: analysis of trajectories over time and associated factors. Child Adolesc Psychiatry Ment Health. (2022) 16:42. doi: 10.1186/s13034-022-00474-x 35689203 PMC9186010

[43] Bersia M Berchialla P Charrier L Lemma P Borraccino A Nardone P . Mental well-being: 2010-2018 trends among Italian adolescents. Int J Environ Res Public Health. (2022) 19:863. doi: 10.3390/ijerph19020863 35055683 PMC8775535

[44] Sampasa-Kanyinga H Lien A Hamilton HA Chaput JP. The Canadian 24-hour movement guidelines and self-rated physical and mental health among adolescents. Can J Public Health Rev Can Sante Publique. (2022) 113:312–21. doi: 10.17269/s41997-021-00568-7 34580829 PMC8975895

