# Supplementary material for: Gender specific mental health among adolescents in Northern Italy: a cross-sectional study
Source: Front Public Health. 2026 Jan 16;13:1705580. doi: 10.3389/fpubh.2025.1705580 (PMC12857638; doi:10.3389/fpubh.2025.1705580)
Supplement: Supplementary file 1 [file Data_Sheet_1.pdf]

1.1 Supplement

Comparing the groups of complete and only sparsely completed questionnaires for parental answers, we found that sparsely completing adolescents were slightly, but significantly (p<0.001) older. All other demographic variables did not significantly differ between the two groups. Even hours of sleep and hours of sports a week did not differ between groups. According to parental answers, the group of sparsely completed questionnaires had significantly (p=0.035) more often an elevated problematic internet use score (GPIUS-2), higher percentages of strongly perceived school burden (p=0.041) and of strongly perceived global burdens (p=0.015) and experienced lower social support (p<0.001).

Table S1 Comparing parental answers for demographic and personal parameters between correctly completed and sparsley completed self-reports

**parameters**	**complete answers N=1,470 Mean+-Standard Deviation**	**Sparsely completed answers N=1,083 Mean+- Standard Deviation**	**p-value***
age	14.38+-2.30	14.49+-2.415	<0.001
hours of sleep	8.23+-0.94	8.30+-0.91	n.s.
female	50.2%	46.7%	n.s.
single parenthood	11.9%	12.7%	n.s.
migration background	8.5%	9.5%	n.s.
urban residence	28.7%	29.2	n.s.
low parental educational level	19.2%	17.9%	n.s.
FAS III			
low	17.7%	15.4%	n.s.
middle	55.8%	57.3%	
high	26.5%	27.3%	
elevated GPIUS2	32.0%	36.1%	0.035
sport 3 times a week	61.2%	61.8%	n.s.
high school burden	36.2%	40.2%	0.041
high global burden	25.6%	30.0%	0.015
MSPSS			
low	11.7%	16.5%	<0.001
moderate	12.8%	15.1%	
high	75.5%	68.4%	

**p-values refer to: Man-Whitney test for age and hours of sleep; Chi-square tests for nominal and ordinal data*
